# *IL-10* Gene Polymorphisms Are Associated with Post-Bronchiolitis Lung Function Abnormalities at Six Years of Age

**DOI:** 10.1371/journal.pone.0140799

**Published:** 2015-10-16

**Authors:** Eero Lauhkonen, Petri Koponen, Johanna Teräsjärvi, Kirsi Gröndahl-Yli-Hannuksela, Juho Vuononvirta, Kirsi Nuolivirta, Jyri O. Toikka, Merja Helminen, Qiushui He, Matti Korppi

**Affiliations:** 1 Tampere Center for Child Health Research, Tampere University and University Hospital, Tampere, Finland; 2 Department of Medical Microbiology and Immunology, Turku University, Turku, Finland; 3 Department of Infectious Disease Surveillance and Control, National Institute for Health and Welfare, Turku, Finland; 4 Seinäjoki Central Hospital, Seinäjoki, Finland; 5 Department of Clinical Physiology, Tampere University Hospital, Tampere, Finland; 6 Department of Clinical Physiology, Turku University Hospital, Turku, Finland; Imperial College London, UNITED KINGDOM

## Abstract

**Aim:**

Interleukin-10 (IL-10) has been associated with wheezing and asthma in children and the genetic variation of the IL-10 cytokine production may be linked to post-bronchiolitis lung function. We used impulse oscillometry (IOS) to evaluate the associations of *IL10* polymorphisms with lung function at a median age of 6.3 years in children hospitalised for bronchiolitis before six months of age.

**Methods:**

We performed baseline and post-exercise IOS on 103 former bronchiolitis patients. Data on single nucleotide polymorphisms (SNP) of *IL10* rs1800896 (–1082G/A), rs1800871 (–819C/T), rs1800872 (–592C/A) were available for 99 children and of *IL10* rs1800890 (–3575T/A) for 98 children.

**Results:**

*IL10* rs1800896, rs1800871 and rs1800872 combined genotype AA+CT+CA and carriage of haplotype ATA, respectively, were associated with higher resistance and lower reactance in baseline IOS in adjusted analyses. At *IL10* rs1800890, the A/A-genotype and carriers of A-allele were associated with lower reactance in baseline IOS. There were no significant associations between the studied SNPs and airway hyper-reactivity to exercise.

**Conclusion:**

Low-IL-10-producing polymorphisms in the IL-10 encoding gene were associated with obstructive lung function parameters, suggesting an important role for IL-10 in development of lung function deficit in early bronchiolitis patients.

## Introduction

It is estimated that 2–3% of each age cohort are hospitalised for viral bronchiolitis during the first year of life. [1] There is increasing evidence that both virus-specific factors [2,3] and genetic variations of innate immunity contribute to later outcomes, including post-bronchiolitis wheezing [4,5] and the development of asthma. [6]

Interleukin-10 (IL-10) is a pivotal regulatory cytokine produced by blood monocytes and alveolar macrophages in the lungs [7] and reduced IL-10 levels have been reported in the alveolar fluid of asthmatics. [8] The *IL-10* gene is highly polymorphic and single nucleotide polymorphisms (SNP) in the proximal promoter region rs1800896, rs1800871 and rs1800872 form distinct haplotypes associated with IL-10 production. [9,10] Furthermore, these polymorphisms have been associated with paediatric asthma. [6,11] In the distal promoter region, carriage of allele A in the *IL10* rs1800890 has been associated with low IL-10 production in experimental studies. [12]

Earlier studies on this cohort of 166 infants hospitalised for bronchiolitis at less than six months of age reported that SNPs in the *IL-10* gene were associated with severe rhinovirus bronchiolitis and post-bronchiolitis asthma. [2,13]

The use of impulse oscillometry (IOS) [14] to measure lung function during tidal breathing is a promising technique for children under the age of seven. [15,16] We have previously published lung function results by IOS at age of five to seven years in 103 children hospitalised for bronchiolitis at less than six months of age. [17] Resistance or reactance at 5 Hz were pathological in 20% of the children compared to Finnish population-based, height-adjusted reference values.

We hypothesised that polymorphisms in the *IL-10* gene could be associated with post-bronchiolitis lung function disorders. This study evaluated the association of polymorphisms in the proximal promoter region *IL10* rs1800896 (–1082G/A), rs1800871 (–819C/T) and rs1800872 (–592C/A) or in the distal promoter region *IL10* rs1800890 (–3575T/A) with IOS results at a median age of 6.3 years in children hospitalised for bronchiolitis before six months of age.

## Patients and Methods

### Design

We prospectively followed up 166 children hospitalised for bronchiolitis before six months of age until they were five to seven years of age [18]. Of these, 127 attended a clinical follow-up visit at a median age of 6.3 years [18] and 103 of the children under the age of seven underwent impulse oscillometry (IOS), including baseline and post-exercise measurements. [17] As previously published [19], weight and height were measured and body mass index (BMI) was calculated and expressed as Z-scores from the population means. One child was excluded from the analyses because of a pathologically low BMI Z-score, corresponding to less than 16 kg/m^2^ in a young adult. Early-life risk factors like maternal asthma, atopic eczema in infancy, maternal smoking during pregnancy and respiratory syncytial virus (RSV) aetiology of bronchiolitis had been collected during the hospitalisation for infant bronchiolitis. [5]

Data on SNPs of *IL10* rs1800896, rs1800871 and rs1800872 were available from 99 of the 103 children included in the IOS study, and of *IL10* rs1800890 were available from 98. The median age of the children was 6.3 years, with a standard deviation of 0.46 years, and 51 (50%) were boys. RSV was identified in 60.2% of cases during bronchiolitis.

### Impulse oscillometry

Impulse oscillometry technique is a modification of the forced oscillations method [14] described in detail elsewhere. [20] The lung function examination is based on the measurement of total airway impedance (Zrs) in response to 5–35Hz oscillations conducted to the bronchial tree through a mouthpiece during quiet breathing. The airways resistance (Rrs) describes the resistive forces to the airflow and reactance (Xrs) reflects the elastic properties of the airway and the surrounding lung tissue, both components being derived from the measured Zrs. Other measured parameters describe the changes in bronchial tone and recoil: the rate of change in the Rrs as a function of the oscillation frequency (dRrs/df) and the point where the resistive and the elastic forces equal each other marks the resonant frequency (Fres). Thus, the main parameters observed are the Rrs and Xrs, where low <15Hz frequencies represent the measurement of more peripheral and >15 Hz more central airways. In a typical peripheral obstructive pattern, the Rrs at 5Hz (Rrs5Hz) rises above normal and the dRrs/df becomes more negative, and due to dynamic loss of lung compliance, the reactance at 5Hz (Xrs5Hz) decreases below normal resulting in increased Fres. IOS is applicable in exercise-challenge testing and bronchodilator testing, where change in resistance at 5Hz more than 35% is considered pathological. [21,22]

### Lung function measurement

We used the Masterscreen IOS (Jaeger, Hochberg, Germany) to obtain baseline measurements, under the supervision of an experienced clinical physiologist (JOT), and these were pre-analysed to be graphically appropriate, free from artefacts and coherent in a set criteria (>0.6 at 5Hz and >0.9 at 10Hz) [15,16]. The post-exercise IOS measurements were taken after the children performed an outdoor, eight-minute free-running exercise challenge test (ECT) at more than 90% of the expected maximal heart rate. The mean IOS values were then transformed to height-adjusted Z-scores. [21]

As published previously, [17] either Rrs5Hz or Xrs5Hz was pathological in the baseline IOS measurements in 21 of the study subjects (20.4%) when compared to Finnish population-based, height-adjusted references. [21] Rrs5Hz was pathological in eight (7.8%) cases and Xrs5Hz was pathological in 19 (18.4%) cases. Only one case showed an irreversible lung function reduction that was not responsive to bronchodilators.

### Genetics

We carried out genotyping of the *IL10* rs1800896 (–1082G/A) SNP using the ABI PRISM 7000 Sequence Detection System for both polymerase chain reaction (PCR) and allelic discrimination. [2] The *IL10* rs1800890 (–3575A/T) genotypes were determined by pyrosequencing as described previously. [23] The detection of the IL10 polymorphism sites, rs1800871 (-819A/G) and rs1800872 (-592T/G), were performed using PCR and sequencing of the amplified region. The SNPs at positions -592 and -819 were detected simultaneously in one PCR reaction. The sequence of PCR primers was: forward 5’-TAGGTCTCTGGGCCTTAGTT-3’ and reverse 5’-AAGGCCAATTTAATCCAAGGTT-3’. The correct PCR product size (440 bp) was verified with agarose gel electrophoresis. The forward primer was used also for the sequencing reaction. Sequencing reactions were performed at the Institute for Molecular Medicine Finland in Helsinki. All PCR reactions were performed in the following conditions: initial denaturation at 95°C, denaturation at 95°C for two minutes, annealing at 60°C for 30 seconds and extension at 72°C for 40 seconds. After 40 cycles, final denaturation was carried out at 72°C for seven minutes. Our test provided the reverse transcription sequence, instead of the forward transcription sequence reported in most previous publications. Therefore, to enable us to compare the results with other studies, we have expressed the alleles G/A (*IL10* rs1800871) and G/T (*IL10* rs1800872) as C/T (*IL10* rs1800871) and C/A (*IL10* rs1800872), respectively.

**Fig 1:**
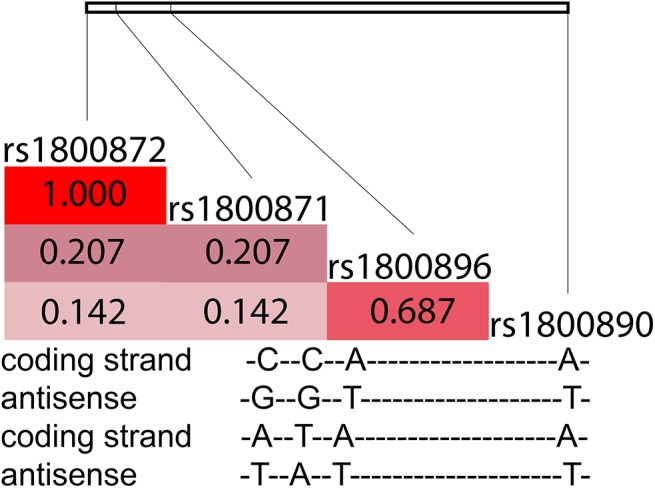
R^2^ -values for the studied *IL-10* polymorphisms in Finnish population based data. *The formation of ACC(A)/ATA(A) genotype from the parental DNA strands described as an example*. *Colour = R* ^*2*^ *-correlation*, *all pairs D´ = 1*. *Modified from* [28].

The measured SNPs were tested to be in HWE (p>0.05) and haplotype frequencies of rs1800896, rs1800871 and rs1800872 were calculated with Haploview 4.2. [24] We compared the SNP minor allele frequencies to population-based public data. [25] The *IL10* rs1800896, rs1800871 and rs1800872 fall in distinct haplotypes in the Caucasian population. [9,26] Individual haplotypes of rs1800896, rs1800871 and rs1800872 were counted from unphased genotype data using the Clark´s algorithm [27] where all homozygote and single-site heterozygotes were identified and haplotypes resolved by direct counting. The SNPs rs1800871 and rs1800872 were co-segregating. This way 87% of the haplotypes were unambiguous, and the rest 13% could be resolved using the haplotype frequencies. All the studied four SNPs are highly linked, (Fig 1) but we were not able to resolve all four allele haplotypes, thus the rs1800890 was left out of the haplotype analyses.

### Statistics

SPSS version 21 (IBM, NY, USA) was used in the statistical analyses of the data. The results are expressed as means and standard deviations (SD) for continuous variables and as numbers and frequencies for categorised variables. Analysis of co-variance (ANCOVA) was used in the analyses of continuous data, when appropriate, adjusted for current age, BMI Z-score, maternal smoking during pregnancy, respiratory syncytial virus (RSV) and rhinovirus aetiology of bronchiolitis.

Firstly, we compared genotypes separately and then we compared carriers of major alleles, for example dominant XX+Xx versus non-carriers xx, and thereafter carriers of minor alleles, for example recessive xx+Xx versus non-carriers XX.

### Ethics

The Ethics Committee of the Tampere University Hospital District approved the study and a written informed consent was obtained from the parents before the children were enrolled as infants and at the clinical control visit. The genetic studies were carried out anonymously.

## Results

The minor allele frequency of rs1800896 was comparable to Finnish and European populations. (Table 1) In rs1800871 and rs1800872 the minor allele frequencies were slightly lower and in rs1800890 somewhat higher compared to Finnish and European populations. (Table 1)

**Table 1:** Minor allele frequencies of the studied *IL-10* polymorphisms and comparison to population genetics.

Single nucleotide polymorphism	Minor allele frequency	FIN	EUR
rs1800896 (G>A)	0.45	0.40	0.45
rs1800871(C>T) /…72 (C>A)	0.18	0.24	0.24
rs1800890 (A>T)	0.39	0.31	0.37

*FIN = Finnish*, *EUR = European as in* [25]

### Single nucleotide polymorphisms

The frequencies and IOS results of the *IL10* rs1800896, rs1800871, rs1800872 and rs1800890 genotypes and allele carriage were first analysed separately.

We found that 26 of the 99 children tested had the G/G genotype of *IL10* rs1800896, 48 had the A/G genotype and 25 had the A/A genotype. There were no significant associations between the genotypes (Table 2) or allele carriage (Table 2) of *IL10* rs1800896 and the IOS results.

**Table 2:** Impulse oscillometry presented as Z-scores in relation to *IL-10* rs1800896 polymorphism in 99 children under the age of seven after hospitalisation for bronchiolitis at less than six months of age.

IOS parameter	G/G	A/G	A/A	p^a^	G-allele	p^a^ *	A-allele	p^a^ *
	n = 26	n = 48	n = 25		n = 74		n = 73	
*Baseline*	Mean (SD)	Mean (SD)	Mean (SD)		Mean (SD)		Mean (SD)	
Rrs5Hz	-0.16 (0.96)	-0.02 (1.11)	-0.05 (1.07)	0.86	-0.07 (1.05)	0.59	-0.03 (1.09)	0.82
Xrs5Hz	-0.76 (1.25)	-0.72 (1.08)	-0.71 (1.46)	0.93	-0.73 (1.13)	0.79	0.71 (1.21)	0.74
Fres	2.32 (0.64)	2.27 (0.87)	2.06 (0.94)	0.67	2.29 (0.79)	0.39	2.20 (0.89)	0.57
dRs/df	-0.86 (1.00)	-1.16 (1.14)	-0.91 (1.19)	0.65	-1.06 (1.10)	0.54	-1.08 (1.16)	0.67
***Post-exercise values***								
Rrs5Hz	0.05 (1.12)	0.34 (1.12)	0.04 (1.25)	0.90	0.24 (1.12)	0.97	0.24 (1.16)	0.68
Xrs5Hz	-1.08 (1.37)	-1.09 (1.37)	-1.01 (1.74)	0.93	-1.09 (1.36)	0.97	-1.07 (1.49)	0.71
Fres	2.48 (0.75)	2.44 (0.87)	2.19 (1.01)	0.69	2.45 (0.83)	0.55	2.35 (0.92)	0.42
dRs/df	-1.20 (1.08)	-1.54 (1.40)	-1.17 (1.61)	0.62	-1.42 (1.30)	0.45	-1.41 (1.47)	0.77

Zrs = Total Impedance; Rrs = Resistance; Xrs = Reactance; Fres = Resonant Frequency; dRrs/df = Frequency Dependency of Resistance

^**a**^adjusted for age, maternal smoking during pregnancy, RSV and rhinovirus aetiology of bronchiolitis and BMI Z-score

*allele carriers vs. non-carriers

Our results showed that 68/99 had the C/C genotype of *IL10* rs1800871/ *IL10* rs1800872, 27 had the C/T or C/A genotype and four had the T/T or A/A genotype. Baseline Xrs5Hz was lowest in children with the T/T (*IL10* rs1800871) or A/A (*IL10* rs1800871) genotypes and highest in those with the C/C genotype. (Table 3) The carriage of major allele C was associated with higher baseline Xrs5Hz (adjusted p = 0.03) and post-exercise Xrs5Hz and Fres (adjusted p = 0.02 and p = 0.05, respectively). The carriage of minor allele T (*IL10* rs1800871) or A (*IL10* rs1800872) was associated with higher Rrs5Hz and lower Xrs5Hz in baseline IOS than non-T or non-A carriers, respectively. (Table 3) There were no significant associations between *IL10* rs1800871/ *IL10* rs1800872 minor alleles T or A carriage, and the post-exercise IOS results.

**Table 3:** Impulse oscillometry presented as Z-scores in relation to *IL-10* rs1800871/rs1800872 polymorphisms in 99 children under the age of seven after hospitalisation for bronchiolitis at less than six months of age.

IOS parameter	C/C	C/T or C/A	T/T or A/A	p^a^	C-allele	p^a^ *	T- /A-allele	p^a^ *
	n = 68	n = 27	n = 4		n = 95		n = 31	
*Baseline*	Mean (SD)	Mean (SD)	Mean (SD)		Mean (SD)		Mean (SD)	
Rrs5Hz	-0.17 (1.00)	0.15 (1.16)	0.27 (1.16)	0.08	-0.08 (1.05)	0.64	0.17 (1.14)	**0.02**
Xrs5Hz	-0.60 (0.98)	-0.85 (1.58)	-2.07 (1.47)	**0.04**	-0.67 (1.18)	**0.03**	-1.01 (1.60)	**0.04**
Fres	2.17 (0.84)	2.32 (0.85)	2.73 (0.63)	0.33	2.21 (0.84)	0.26	2.37 (0.82)	0.20
dRs/df	-0.95 (1.00)	-1.12 (1.38)	-1.48 (1.13)	0.33	-1.00 (1.12)	0.37	-1.17 (1.34)	0.16
***Post-exercise values***								
Rrs5Hz	0.09 (1.14)	0.29 (1.13)	1.11 (1.34)	0.11	0.15 (1.13)	0.11	0.40 (1.17)	0.07
Xrs5Hz	-1.02 (1.36)	-0.93 (1.60)	-2.70 (1.50)	0.07	-1.00 (1.43)	**0.02**	-1.16 (1.67)	0.39
Fres	2.33 (0.91)	2.41 (0.74)	3.20 (1.06)	0.13	2.35 (0.86)	**0.05**	2.51 (0.81)	0.22
dRs/df	-1.29 (1.36)	-1.38 (1.46)	-2.34 (0.94)	0.25	-1.32 (1.38)	0.13	-1.50 (1.43)	0.25

Zrs = Total Impedance; Rrs = Resistance; Xrs = Reactance; Fres = Resonant Frequency; dRrs/df = Frequency Dependency of Resistance

^**a**^adjusted for age, maternal smoking during pregnancy, RSV and rhinovirus aetiology of bronchiolitis and BMI Z-score

*allele carriers vs. non-carriers

With regard to *IL10* rs1800890, 35/98 had the A/A genotype, 50 (51%) the A/T genotype and 13 (13%) had the T/T genotype. Baseline Xrs5Hz was highest in children with the T/T genotype and lowest in those with the A/A genotype. (Table 4) The carriage of the major allele A was associated with lower baseline Xrs5Hz (adjusted p = 0.03) when compared to the non-carriers. (Table 4) The carriage of the minor allele T was associated with lower baseline Rrs5Hz (adjusted p = 0.04, respectively) and higher Xrs5Hz (adjusted p = 0.04) and lower post-exercise Rrs5Hz (adjusted p = 0.03). (Table 4)

**Table 4:** Impulse oscillometry presented as Z-scores in relation to *IL-10* rs1800890 polymorphism in 98 children under the age of seven after hospitalisation for bronchiolitis at less than six months of age.

IOS parameter	A/A	A/T	T/T	p^a^	A-allele	p^a^ *	T-allele	p^a^ *
	n = 35	n = 50	n = 13		n = 85		n = 74	
*Baseline*	Mean (SD)	Mean (SD)	Mean (SD)		Mean (SD)		Mean (SD)	
Rrs5Hz	0.10 (1.03)	-0.17 (1.10)	-0.22 (0.77)	0.13	-0.06 (1.08)	0.58	-0.18 (1.04)	**0.04**
Xrs5Hz	-1.02 (1.25)	-0.64 (1.11)	-0.06 (1.08)	**0.03**	-0.79 (1.18)	**0.03**	-0.52 (1.12)	**0.04**
Fres	2.38 (0.85)	2.16 (0.84)	2.02 (0.66)	0.20	2.25 (0.85)	0.36	2.13 (0.81)	0.08
dRs/df	-1.23 (1.09)	-0.93 (1.14)	-0.64 (0.93)	0.20	-1.06 (1.12)	0.24	-0.87 (1.10)	0.10
***Post-exercise values***								
Rrs5Hz	0.37 (1.12)	0.09 (1.20)	-0.07 (0.85)	0.10	0.20 (1.17)	0.37	0.05 (1.13)	**0.03**
Xrs5Hz	-1.27 (1.53)	-1.06 (1.44)	-0.33 (0.90)	0.11	-1.14 (1.47)	0.06	-0.91 (1.37)	0.15
Fres	2.49 (0.78)	2.36 (0.92)	2.04 (0.79)	0.12	2.41 (0.86)	0.12	2.30 (0.90)	0.09
dRs/df	-1.63 (1.49)	-1.26 (1.30)	-0.83 (1.12)	0.16	-1.41 (1.39)	0.19	-1.17 (1.27)	0.08

Zrs = Total Impedance; Rrs = Resistance; Xrs = Reactance; Fres = Resonant Frequency; dRrs/df = Frequency Dependency of Resistance

^**a**^ adjusted for age, maternal smoking during pregnancy, RSV and rhinovirus aetiology of bronchiolitis and BMI Z-score

*allele carriers vs. non-carriers

### Combined genotypes and haplotypes

The three bi-allelic polymorphisms rs1800896, rs1800871 and rs1800872 in the proximal promoter region of *IL-10* gene were analysed as combined genotypes and according to haplotype carriage.

Table 5 summarises the frequencies of the combined genotypes and haplotype frequencies of the *IL10* rs1800896, rs1800871 and rs1800872 genes. The haplotype GCC (major alleles) was present in 71.7%, ACC was present in 59.6%, ATA (minor alleles) was present in 21.2% and GTA was present in 10.1%.

**Table 5:** Combined *IL-10* rs1800896, rs1800871 and rs1800872 genotypes and haplotype frequencies of 99 children hospitalised for bronchiolitis in infancy.

**Genotype rs1800896+rs1800871+rs1800872 (haplotype)**	n (%)
GG+CC+CC (GCC/GCC)	16 (16.2)
GG+CT+CA (GCC/GTA or GTA/GCC)	7 (7.1)
GG+TT+AA (GTA/GTA)	3 (3.0)
GA+CC+CC (GCC/ACC or ACC/GCC)	35 (35.4)
GA+CT+CA (GCC/ATA or ATA/GCC)*	13 (13.1)
GA+TT+AA (GTA/ATA or ATA/GTA)	0 (0.0)
AA+CC+CC (ACC/ACC)	17 (17.2)
AA+CT+CA (ACC/ATA or ATA/ACC)	7 (7.1)
AA+TT+AA (ATA/ATA)	1 (1.0)
**Haplotype frequency**	f
GCC	0.44
GTA	0.07
ACC	0.39
ATA	0.11

*GTA/ACC or ACC/GTA not present as estimated from the study population frequencies.

The genotype AA+CT+CA was significantly associated with higher baseline Rrs5Hz and lower baseline Xrs5Hz than the non-AA+CT+CA genotypes. (Table 6) The genotype GG+TT+AA was significantly associated with lower baseline and post-exercise Xrs5Hz. (Table 6)

**Table 6:** Impulse oscillometry presented as Z-scores in relation to combined *IL-10* rs1800896, rs1800871 and rs1800872 genotypes in 99 children under the age of seven after hospitalisation for bronchiolitis in infancy.

IOS parameter	GG+CC+CC	GG+CT+CA	GG+TT+AA	GA+CC+CC	GA+CT+CA	AA+CC+CC	AA+CT+CA
	n = 16	n = 7	n = 3	n = 35	n = 13	n = 17	n = 7
*Baseline*	mean (SD)	mean (SD)	mean (SD)	mean (SD)	mean (SD)	mean (SD)	mean (SD)
Rrs5Hz	-0.20 (0.57)	-0.30 (1.47)	0.40 (1.38)	-0.06 (1.11)	0.09 (1.14)	-0.36 (1.09)	**0.72 (0.68)** ^**a**^
Xrs5Hz	-0.70 (1.15)	-0.14 (0.75)	**-2.52 (1.42)** ^**a**^	-0.66 (0.95)	-0.86 (1.39)	-0.36 (0.88)	**-1.55 (2.30)** ^**a**^
Fres	2.23 (0.58)	2.29 (0.73)	2.89 (0.66)	2.27 (0.88)	2.29 (0.86)	1.91 (0.93)	2.39 (1.03)
dRs/df	-0.87 (0.91)	-0.56 (1.06)	-1.55 (1.37)	-1.11 (1.12)	-1.31 (1.40)	-0.72 (1.00)	-1.32 (1.64)
***Post-exercise values***							
Rrs5Hz	-0.19 (0.83)	0.11 (1.33)	1.20 (1.63)	0.36 (1.15)	0.29 (1.09)	-0.19 (1.29)	0.49 (1.14)
Xrs5Hz	-0.82 (1.15)	-0.78 (1.26)	**-3.15 (1.48)** ^**a**^	-1.03 (1.29)	-1.26 (1.59)	-1.21 (1.71)	-0.47 (1.97)
Fres	2.31 (0.63)	2.48 (0.58)	3.40 (1.20)	2.41 (0.96)	2.51 (0.63)	2.19 (1.04)	2.13 (1.06)
dRs/df	-1.04 (0.90)	-0.99 (1.26)	-2.54 (1.04)	-1.50 (1.44)	-1.64 (1.34)	-1.09 (1.55)	-1.28 (1.96)

Zrs = Total Impedance; Rrs = Resistance; Xrs = Reactance; Fres = Resonant Frequency; dRrs/df = Frequency Dependency of Resistance

^**a**^adjusted p<0.05 vs. non-AA+CT+CA (n = 92) or non-GG+TT+AA (n = 96), as adjusted for age, maternal smoking during pregnancy, RSV- and rhinovirus etiology of bronchiolitis and BMI Z-score

The carriage of haplotype ATA, consisting of three minor alleles, was associated with higher baseline Rrs5Hz. (Table 7)

**Table 7:** Impulse oscillometry presented as Z-scores in relation to *IL-10* rs1800896, rs1800871 and rs1800872 haplotype carriage in 99 pre-school-aged children after hospitalization for bronchiolitis in infancy.

IOS parameter	GCC	GTA	ACC	ATA	p^a^
	n = 71	n = 10	n = 59	n = 21	
*Baseline*	Mean (SD)	Mean (SD)	Mean (SD)	Mean (SD)	
Rrs5Hz	-0.09 (1.04)	-0.09 (1.40)	-0.05 (1.09)	0.29 (1.01)	**0.03**
Xrs5Hz	-0.66 (1.07)	-0.86 (1.46)	-0.68 (1.19)	-1.08 (1.69)	0.07
Fres	2.27 (0.79)	2.47 (0.73)	2.18 (0.91)	2.32 (0.88)	0.44
dRs/df	-1.04 (1.09)	-0.86 (1.18)	-1.02 (1.11)	-1.32 (1.41)	0.10
***Post-exercise values***					
Rrs5Hz	0.20 (1.09)	0.43 (1.43)	0.22 (1.20)	0.38 (1.06)	0.24
Xrs5Hz	-1.00 (1.30)	-1.49 (1.69)	-1.02 (1.49)	-1.00 (1.69)	0.98
Fres	2.41 (0.79)	2.76 (0.86)	2.31 (0.98)	2.39 (0.78)	0.81
dRs/df	-1.37 (1.29)	-1.46 (1.34)	-1.36 (1.52)	-1.53 (1.50)	0.39

Zrs = Total Impedance; Rrs = Resistance; Xrs = Reactance; Fres = Resonant Frequency; dRrs/df = Frequency Dependency of Resistance

^**a**^ATA vs. non-ATA (n = 78) adjusted for age, maternal smoking during pregnancy, RSV- and rhinovirus etiology of bronchiolitis and BMI z-score

## Discussion

The main results of this post-bronchiolitis lung function study at a median age of 6.3 years were that *IL10* polymorphisms rs1800896, rs1800871, rs1800872 and rs1800890 were associated with lung function measured with IOS. Low IL-10 producing polymorphisms were associated with obstructive IOS baseline results. There were no significant associations between *IL10* polymorphisms and airway hyper-reactivity in response to exercise. Although some knowledge has accumulated on the association between cytokines or cytokine genetics, such as *IL-10* polymorphisms, and wheezing or asthma in preschool-aged children, no lung function studies have so far been published.

IL-10 is a key regulator of human immune responses and possesses many immunosuppressive functions that protect the host from abnormally strong inflammation and subsequent tissue damage playing a protective role in allergic disease. [7] The other side of the coin is that the suppression of immune responses may lead to more severe infections that last longer. For example, *IL-10* gene polymorphisms have been associated with severe RSV and rhinovirus bronchiolitis and with post-infectious wheezing in childhood. [2,4] In experimental studies, IL-10-deficient mice developed allergic responses but airway reactivity did not increase in response to allergen challenge. [29]

The studied *IL-10* gene polymorphisms are functional as they regulate IL-10 production. In rs1800890, the presence of allele A has been associated with low IL-10 production [12] and, similarly in rs1800896, rs1800871 and rs1800872, the haplotype ATA has been associated with low IL-10 levels. [10] An earlier study stated that the presence of allele A at *IL10* rs1800896 (G/A) was associated with lower IL-10 production regardless of the alleles at rs1800871 or rs1800872. [9] However, another study suggested that only the ACC haplotype was associated with high IL-10 levels. [26]

In this study, the presence of *IL10* rs1800896, rs1800871 and rs1800872 haplotype ATA was associated with increased resistance in the baseline IOS measurements. Our findings are in line with a study of 518 asthmatic children at a mean age of 8.1 years, which reported that haplotype ATA was associated with low forced expiratory volume in one second (FEV1) and that haplotype GCC was associated with high FEV1. [11] In addition, the AA+CT+CA genotype was associated with abnormal baseline IOS results. This genotype includes two *IL10* rs1800896 minor A alleles and the ATA haplotype, which both have a known association with low IL-10 production. [10]

Previously in this cohort, the *IL10* rs1800896 A/A genotype was associated with non-RSV aetiology of bronchiolitis [2], and under the age of seven, the G/G genotype was protective for post-bronchiolitis asthma. Correspondingly, the presence of allele A increased the risk of asthma [13]. In the current study, SNP at rs1800896 did not show any significant association with lung function measurements, but the minor allele A was present in the combined genotype and haplotype associated with reduced IOS results.

Data on the *IL-10* rs1800890 polymorphisms were now available in this study, though not possible to be included in the haplotype analyses. The presence of the major allele A was associated with post-bronchiolitis lung function disorders under the age of seven. These findings suggest that *IL10* polymorphisms play a role in the pathogenesis of post-bronchiolitis lung dysfunction, likely via differences in IL-10 production during bronchiolitis.

The SNP distribution in *IL10* rs1800896 was not different from the Finnish blood donors in an earlier study [2] and the SNP distributions of *IL10* rs1800871, rs1800872 and rs1800890 were comparable with those of Finnish and European populations. (see Table 1) Although GTA haplotypes are uncommon in Caucasian populations [9,26] their frequency was 0.07 in our study. In previous studies this haplotype has been identified in Dutch population with a frequency of 0.01 [26] and in Asian (Hong Kong) population with a frequency of 0.04. [30] Thus this haplotype is overrepresented in our study population, which could be explained with different, maybe more eastern ancestral background of the Finnish population compared to other European populations.

The main weakness of the present study was the somewhat small number of patients for a genetic study. Due to this, the power of the study was not enough for stratified analyses and it carried a risk of type-two statistical errors. However, the design and data of the study allowed adjusted analyses, which increase the reliability of the revealed associations. The findings were robust to adjustments for the most important confounders like maternal smoking, atopic eczema in infants and viral aetiology of infant bronchiolitis. On the other hand, multiple analyses of different IOS parameters from the same data mean a risk of type-one statistical error. Any control groups with sufficient IOS and genetic data were not available, which is a clear limitation of our study. However, national population-based age-specific and height-adjusted reference values were available for the IOS measurements [21]. We generated IOS measurements of good-quality for more than 96% of the study subjects, and genetic data were available for 92% of them.

The results of this study provide additional evidence that lung function decline measured by IOS in former bronchiolitis patients under the age of seven may be associated with low-producing genotypes and haplotypes of the IL-10 encoding gene polymorphisms at rs1800896, rs1800871, rs1800872 and rs1800890. This preliminary evidence of cytokine-associated lung function disorder after viral bronchiolitis might reflect the impact of the infection itself during the first months of life or result from a predisposition to a subsequent disease processes in individuals with low IL-10 production.
